# Identification of a Novel Biomarker Panel for Breast Cancer Screening

**DOI:** 10.3390/ijms252111835

**Published:** 2024-11-04

**Authors:** Maria Vaida, Kamala K. Arumalla, Pavan Kumar Tatikonda, Bharadwaj Popuri, Rashid A. Bux, Paramjit S. Tappia, Guoyu Huang, Jean-François Haince, W. Randolph Ford

**Affiliations:** 1 Department of Analytics, Harrisburg University of Science and Technology, Harrisburg, PA 17101, USA; mvaida@harrisburgu.edu (M.V.); karumalla@my.harrisburgu.edu (K.K.A.); ptatikonda@my.harrisburgu.edu (P.K.T.); bpopuri@my.harrisburgu.edu (B.P.); marriotts2010@gmail.com (W.R.F.); 2 BioMark Diagnostics Inc., Richmond, BC V6X 2W2, Canada; rahmed@biomarkdiagnostics.com; 3 Asper Clinical Research Institute, Winnipeg, MB R2H 2A6, Canada; 4 BioMark Diagnostic Solutions Inc., Quebec City, QC G1P 4P5, Canada; ghuang@biomarkdiagnostics.com (G.H.); jhaince@biomarkdiagnostics.com (J.-F.H.)

**Keywords:** breast cancer, biomarkers, metabolomic profiling, early detection, screening, machine learning

## Abstract

Breast cancer remains a major public health concern, and early detection is crucial for improving survival rates. Metabolomics offers the potential to develop non-invasive screening and diagnostic tools based on metabolic biomarkers. However, the inherent complexity of metabolomic datasets and the high dimensionality of biomarkers complicates the identification of diagnostically relevant features, with multiple studies demonstrating limited consensus on the specific metabolites involved. Unlike previous studies that rely on singular feature selection techniques such as Partial Least Square (PLS) or LASSO regression, this research combines supervised and unsupervised machine learning methods with random sampling strategies, offering a more robust and interpretable approach to feature selection. This study aimed to identify a parsimonious and robust set of biomarkers for breast cancer diagnosis using metabolomics data. Plasma samples from 185 breast cancer patients and 53 controls (from the Cooperative Human Tissue Network, USA) were analyzed. This study also overcomes the common issue of dataset imbalance by using propensity score matching (PSM), which ensures reliable comparisons between cancer and control groups. We employed Univariate Naïve Bayes, L2-regularized Support Vector Classifier (SVC), Principal Component Analysis (PCA), and feature engineering techniques to refine and select the most informative features. Our best-performing feature set comprised 11 biomarkers, including 9 metabolites (SM(OH) C22:2, SM C18:0, C0, C3OH, C14:2OH, C16:2OH, LysoPC a C18:1, PC aa C36:0 and Asparagine), a metabolite ratio (Kynurenine-to-Tryptophan), and 1 demographic variable (Age), achieving an area under the ROC curve (AUC) of 98%. These results demonstrate the potential for a robust, cost-effective, and non-invasive breast cancer screening and diagnostic tool, offering significant clinical value for early detection and personalized patient management.

## 1. Introduction

Internationally, breast cancer continues to be the primary cause of mortality in women, surpassing both lung and skin cancers. The American Society of Clinical Oncology (2024) predicts that there will be 297,790 new instances of invasive breast cancer and 55,720 cases of non-invasive breast cancer in the United States in 2024. Additionally, 2800 cases are expected to be detected in men. Furthermore, there are currently more than 3.8 million women who are either living with or have survived this disease [1]. Early identification is essential for successful therapy and possibly halting the advancement of the disease. However, existing techniques such as mammography, which are mostly advised for women between the ages of 40 and 75, have several drawbacks. False positives, a common issue in lung cancer screening, also afflict mammography, resulting in unneeded biopsies, stress, discomfort, and radiation exposure [2]. Even with the application of machine learning models to mammography images, a review of 38 studies involving 68 models has demonstrated that they have not consistently achieved an AUC above 90% [3]. In addition, excluding younger or older individuals from routine cancer screenings risks missing a significant number of cancer cases. The invasive nature of the biopsies currently employed to detect and analyze tumor biomarkers highlights the critical need for non-invasive methods for early-stage breast cancer detection. Such advancements could expand breast cancer screening to people across all risk groups, even those outside typical age ranges for screening, possibly reducing deaths linked to detecting cancer in later stages [4].

Metabolites, the small biomolecules that act as distinct chemical markers of metabolic activities, have great potential to enhance the accuracy and precision of breast cancer screening and early diagnosis. Alteration of the metabolomic profile of an individual often arises from a change in a gene, whether that be a gene mutation, over-expression, or downregulation, and these changes eventually could facilitate cancer development [5]. Metabolites are also closely linked to the phenotype of an organism, which can have a significant impact on the human health [6]. Considering that breast cancer is a highly complex and heterogeneous disease with varying clinical presentations and responses to therapy, metabolomic profiling offers a valuable avenue of measuring changes in a patient’s phenotype [5]. This approach could further enhance our understanding of disease progression and contribute to more precise diagnostic and therapeutic strategies.

While univariate and multivariate analyses of metabolite datasets have shown promise, the need for more robust approaches has been highlighted [7]. Through the utilization of machine learning (ML) and deep learning (DL) algorithms on metabolomics data, it is possible to conceive the creation of models that may identify breast cancer and potentially different subtypes of cancers even prior to the manifestation of symptoms. Comprehensive metabolite profiling offers important perspectives on the fundamental processes driving cancer cell growth. Breast cancer cells exhibit altered metabolic profiles that indicate their increased energy demands, enhanced proliferation, and capacity to avoid programmed cell death [8]. These alterations are observed in the levels of metabolites associated with many processes, including immune suppression, lipid metabolism, and amino acid metabolism. Immune suppressors, such as elevated Kynurenine levels and depleted Tryptophan, play a crucial role in cancer progression by allowing tumors to evade immune detection while promoting their growth and survival [9]. Lipid metabolism plays a crucial role in synthesizing fatty acids. These processes support cell membrane formation and cellular communication but can also lead to the growth of breast cancer by modifying key lipid types like sphingolipids, phospholipids, and fatty acids, which are involved in tumor progression [10,11]. Heightened utilization of glutamine for anabolic functions and altered amino acid compositions responsible for protein synthesis and degradation stimulate the metabolic pathways of amino acids such as Asparagine [12]. The analysis of these metabolic signatures provides a comprehensive overview of cellular processes and physiological states, offering valuable insights into the distinct energy requirements and vulnerabilities of cancer cells. This metabolomic approach has the potential to detect the subtle biochemical alterations associated with early-stage malignancies, thereby presenting a non-invasive and potentially more sensitive screening method. Such a technique could be suitable for application across a wider spectrum of age groups, enhancing our ability to detect and characterize cancer at earlier stages. By elucidating the unique metabolic profiles of breast cancer, this methodology not only contributes to our understanding of cancer biology but also holds promise for improving early detection. 

**Figure 1:**
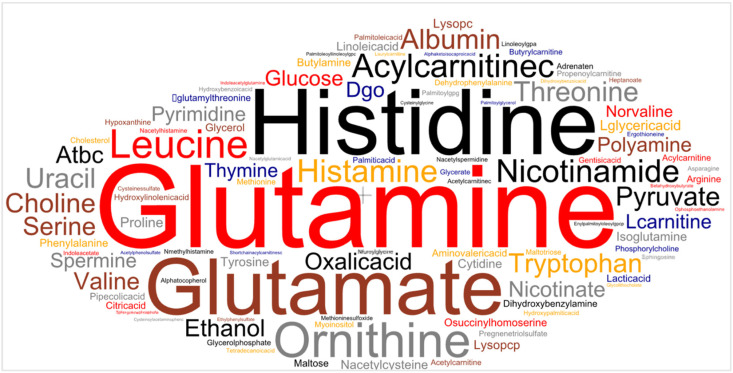
Word cloud of significant metabolites identified as significant indicators of breast cancer in the reviewed breast cancer literature [13,14,15,16,17,18,19,20,21,22,23,24,25,26]. Text size represents the prevalence of the biomarkers in the reviewed literature. The word cloud was generated using the *wordcloud* Python library [27].

While over 100 potential biomarkers have been proposed in the existing literature [13,14,15,16,17,18,19,20,21,22,23,24,25,26] as statistically significant indicators of breast cancer, few are consistently replicated across multiple studies. Among the most important metabolites repeatedly mentioned in the literature are glutamine, histidine, glutamate, ornithine, oxalic acid, threonine, histamine, albumin, pyruvate, nicotinamide, tryptophan, and leucine. Figure 1 displays the frequency at which each metabolite is identified as a significant indicator of breast cancer. The variability in the specific metabolites identified across different studies may be attributed to differences in study populations, the specific biological sample or tissue from which biomarkers are extracted, analytical techniques, and machine learning approaches. Nonetheless, the existing body of research on metabolomic biomarkers for breast cancer detection demonstrates the potential of this approach.

The principal aim of this research is to construct robust, parsimonious, and interpretable models for early-stage breast cancer detection utilizing strategically selected, information-rich metabolomic signatures. Unlike previous studies that rely on singular feature selection techniques such as PLS or LASSO regression, this research combines supervised (SVC, Naive Bayes) and unsupervised (PCA) methods, offering a more robust and interpretable approach to feature selection [13,14,15,16,19,22,26,28,29]. Additionally, this study overcomes the common issue of dataset imbalance by using propensity score matching (PSM), which ensures reliable comparisons between cancer and control groups. The inclusion of a random sampling strategy further enhances the reliability of the selected biomarker sets, leading to a model that achieves an AUC of 98%. This approach has the potential to offer a safer, more personalized, and readily accessible alternative to established screening methods.

## 2. Results

We developed a comprehensive machine learning framework that integrates both supervised and unsupervised feature selection techniques to identify optimal feature sets for breast cancer prediction from a large pool of 132 biomarkers, 8 demographic variables, and 1 biomarker ratio. Among the 132 biomarkers included are Tryptophan, Kynurenine, Glutamine, Methionine, Valine, LysoPC a C16:0, Citric acid, and Creatinine. Given the dataset’s high dimensionality, reducing the number of features was critical to prevent overfitting and enhance model generalization. By systematically narrowing down the feature set, we addressed the challenge posed by the high dimensionality and relatively small sample size, ensuring the models remained both interpretable and robust in predicting breast cancer outcomes.

Another key challenge in this study was the significant imbalance in the dataset, consisting of 185 cancer cases and 53 control cases. To mitigate this issue, we employed propensity score matching. Propensity scores, which estimate the probability of each subject belonging to the cancer group based on covariates, were calculated using a Random Forest (RF) classifier composed of 100 decision trees. The model builds trees on various sub-samples of the dataset and uses averaging to improve predictive accuracy and control overfitting. Each tree uses the best split strategy to optimize classification. The Random Forest algorithm was selected due to its robustness in handling complex data structures and interactions between covariates. This methodology has also been used in breast cancer metabolomics by [17,30,31], demonstrating its effectiveness in handling complex data structures and uncovering key metabolic signatures associated with breast cancer progression. Using the propensity scores generated by the RF model, a Nearest-Neighbor matching algorithm was applied for the matching process, ensuring that each cancer case was paired with the most similar control based on their propensity scores. Specifically, for each treated case, the nearest three control cases (n_neighbors = 3) were selected. The result of the matching process was a dataset where treated individuals and their matched controls shared similar characteristics based on their propensity scores. This matching process resulted in 46 additional controls being identified, creating a final dataset consisting of 185 cancer cases and 99 matched controls (53 original controls and 46 additional matched controls). This step helps ensure that the comparison between the treated and control groups is based on a more balanced set of covariates, thus mimicking a randomized controlled trial. PSM provided a stronger foundation for further machine learning analysis by reducing the bias introduced by the imbalance [32].

With the matched dataset, we developed a Support Vector Classifier (SVC) to refine the feature set and build a predictive model for breast cancer classification. A grid search was performed to tune hyperparameters, including the regularization parameter C (ranging from 0.005 to 5), kernel functions (linear, polynomial, radial basis function (RBF), and sigmoid), and gamma (γ) settings (scale and auto). The C parameter controls the trade-off between maximizing the margin and minimizing classification errors, where a higher value of C results in a more complex model that aims to classify all training points correctly, while a lower value results in a simpler, more generalized model. The kernel function defines the decision boundary by transforming the input data into higher-dimensional space. The RBF kernel is particularly effective for non-linear classification problems, such as those in breast cancer prediction. Gamma (γ) defines the influence of individual data points, where higher values focus on nearby points and lower values allow for a more global perspective. Gamma = ‘scale’ uses a heuristic based on the number of features, ensuring an optimal range for γ. Class weight was set to ‘balanced’ to compensate for any imbalance in the dataset [33,34]. The grid search aimed to balance model complexity and margin separation between the cancer and control groups. The model’s performance was assessed using a variety of metrics, including accuracy, AUC, Matthew’s correlation coefficient (MCC), precision, recall, and F1 score. To validate the model’s robustness, Repeated Stratified k-fold cross-validation was employed with 10 folds, repeated 50 times. The optimal SVC configuration included a regularization parameter C of 5, an RBF kernel, γ set to scale, and a balanced class weight. 

The feature selection methodology combines various ML and statistical sampling approaches to ensure model robustness, interpretability, and generalizability. Our original dataset of 141 features was refined using a combination of three feature selection techniques: Support Vector Classifier, Principal Component Analysis, and Naive Bayes (NB). First, Univariate NB was applied to identify the top 15 features, leveraging accuracy performance across multiple iterations of k-fold cross-validation. A Gaussian Naive Bayes classifier was trained on 100 splits of the data, and the performance metrics—accuracy, F1 score, precision, and recall—were averaged to select the most relevant features. Second, a Linear SVC with L2 regularization, a regularization parameter C of 0.001, and dual formulation was used to select the important features. The model automatically chose 59 features based on their importance as determined by the coefficients of the fitted model. Finally, PCA with eight components was employed, identifying 50 features. The features were selected based on their loadings, representing the variables with the greatest influence on the principal components. Each of these methods contributed a distinct perspective on feature importance, based on accuracy, model coefficients, and variance explained.

We then explored the relationships between these feature sets by calculating unions and intersections. The intersection set highlighted unique features identified across the three models, whereas the union set aggregated robust features that were consistently selected by multiple models. The union of SVC and PCA (SVC ∪ PCA) produced 92 features, while combining SVC with NB (SVC ∪ NB) resulted in a set of 67 features. Similarly, the union of PCA and NB (PCA ∪ NB) yielded 50 features. 

Additionally, we examined the intersections between the methods. SVC and PCA shared 17 common features (SVC ∩ PCA), SVC and NB shared 7 features (SVC ∩ NB), and PCA and NB shared 15 features (PCA ∩ NB). Further analysis of the intersection across all three methods (SVC ∩ PCA ∩ NB) revealed 25 features that were consistently identified across all feature selection techniques, highlighting the robustness of these features. To further explore the uniqueness of each method, we calculated the symmetric differences between feature sets. The difference between SVC and PCA (SVC Δ PCA) identified 75 unique features, while the difference between SVC and NB (SVC Δ NB) identified 60 unique features. The comparison between PCA and NB (PCA Δ NB) yielded 35 features that were distinct to either method. Finally, we aggregated the results by considering the union across all three methods (SVC ∪ PCA ∪ NB), which produced a comprehensive feature set of 92 features. Each of these feature sets were evaluated using the optimized SVC pipeline with Repeated Stratified k-fold cross-validation.

To optimize the combination of features further, we implemented a random sampling strategy over the union set SVC ∪ PCA ∪ NB. Over 15,000 iterations, between 9 and 12 features were randomly selected and evaluated using the optimized SVC pipeline, applying Repeated Stratified k-fold cross-validation with 10 splits and 50 repeats. Out of the 15,000 iterations, 14 resulted in a mean AUC greater than 95%. These high-performing feature sets were consolidated into a list of 34 unique biomarkers, from which a core set of 12 biomarkers appeared in at least four different feature sets during the sampling process. These 12 features demonstrated strong predictive performance, achieving an AUC of 98%, an accuracy of 94%, and an MCC score of 89%. To ensure the robustness of the 12-biomarker feature set, we applied leave-one-out cross-validation (LOOCV) and identified the best performing datasets with 11 and 10 features respectively. The set of 11 biomarkers included SM(OH) C22:2, SM C18:0, C0, C3OH, C14:2OH, C16:2OH, LysoPC a C18:1, PC aa C36:0, Asparagine, the Kynurenine-to-Tryptophan ratio, and age. The 12-feature set added SM(OH) C16:1, and the 10-feature set excluded PC aa C36:0.

**Figure 2:**
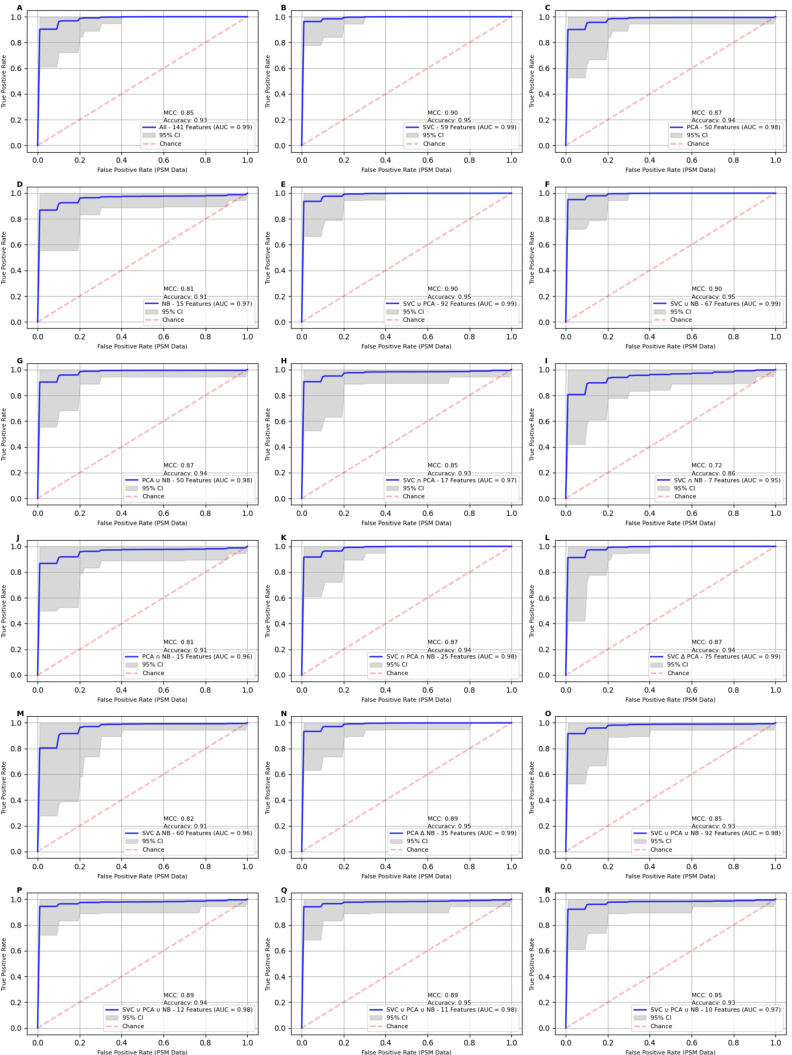
Receiver Operating Characteristic (ROC) curves for classification models on propensity-score-matched (PSM) data using different feature sets. The Matthew’s correlation coefficient (MCC), accuracy, and Area Under the Curve (AUC) values are reported within each panel to quantify the overall model performance. Panels **A**–**R** display AUC curves for the 18 feature sets described in Table 1. The best performance across all models is observed in Panel **B** using the 59 features identified by SVC feature selector, with an AUC of 99%, an MCC score of 91%, and an accuracy of 96%. The 11-feature set (Panel **Q**) scores a point lower on AUC and accuracy and 2 values lower for MCC score, while reducing the number of features 5-fold.

The classification models trained on the propensity-score-matched data exhibited strong predictive performance across various feature sets based on the results shown in Figure 2 and the accompanying performance metrics in Table 1. The SVC feature set (59 features) yielded an AUC of 99%, with an accuracy of 95%, MCC of 90%, and F1 score of 95%. Similarly, the unions of SVC and PCA (92 features) and SVC and NB (67 features) also performed exceptionally well, achieving the same AUC of 99% with an accuracy of 95%, MCC of 90%, and F1 score of 95%. Notably, the 11-feature set also emerged as a strong performer, attaining an AUC of 98%, accuracy of 95%, MCC of 89%, and F1 score of 94%. Further reducing the dataset to 10 features reduced the AUC by 1% and accuracy by 2%. These findings indicate that the 11-feature set strikes an optimal balance between model simplicity and predictive performance, matching the accuracy and precision of the best-performing SVC feature set. Although it has a slightly lower MCC value (89%), AUC (98%), and F1 score (94%), it remains a highly effective and valuable subset for breast cancer prediction as the number of features in this subset decreased five-fold when compared with the SVC feature set.

**Figure 3:**
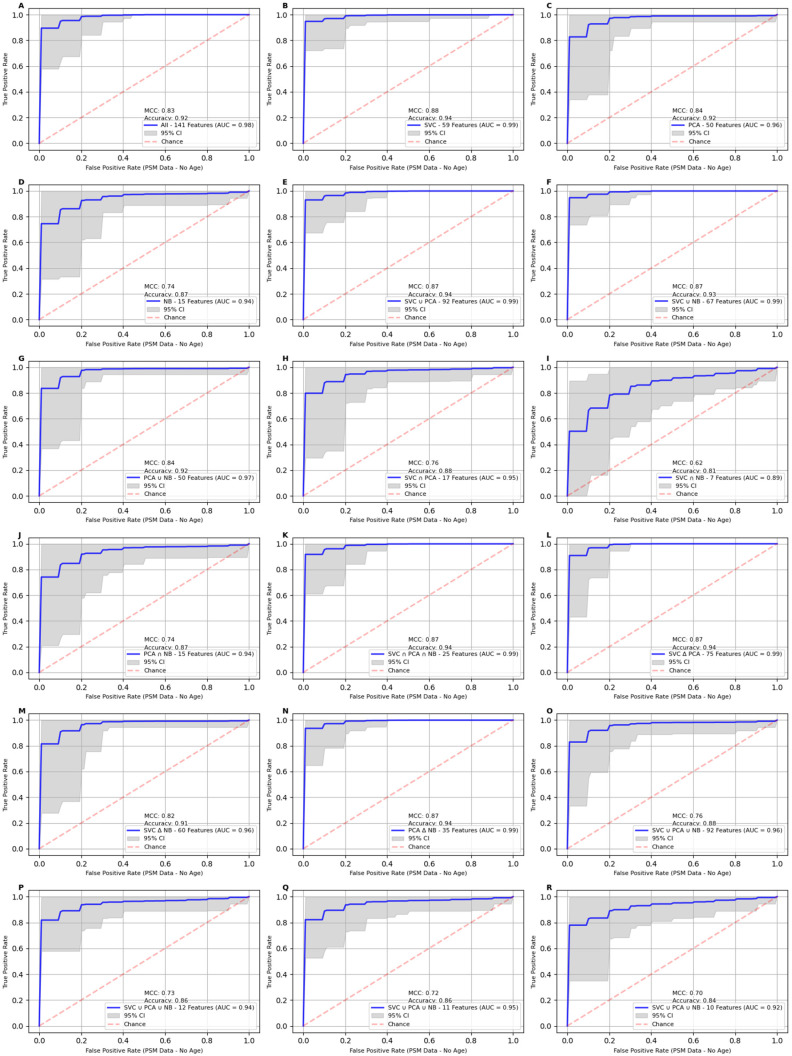
Receiver Operating Characteristic (ROC) curves for classification models on age-excluded propensity-score-matched (PSM) data using different feature sets. The Receiver Operating Characteristic (ROC) curves for classification models applied to PSM feature sets after excluding the age variable. Panels **A**–**R** display AUC curves for the 18 feature sets described in Table 2. Panels **B**, **E**, **L**, **K**, and **N** consistently maintained an accuracy of 94% and an AUC of 99%. However, when the feature set size was reduced to fewer than 12 features, the removal of age had a significant impact on performance. Specifically, the AUC for the 11-feature set (Panel **Q**) decreased from 99% to 94%, with accuracy dropping by 3 percentage points. The MCC showed the most substantial decline, decreasing from 89% to 72%.

To assess the impact of propensity score matching, we applied the model to the original, unmatched dataset. The results revealed a noticeable decline in performance across all metrics across all feature sets, highlighting the critical role of propensity score matching in mitigating dataset imbalance. The model performance decreased across the board, with the full feature set (141 features) achieving an AUC of 93%, accuracy of 87%, and MCC of 63%. The reduced 11-feature set outperformed the full feature set, with an AUC of 95%, accuracy of 90%, and MCC of 72%. In line with the PSM results, the 59 features selected by the SVC set demonstrated the strongest performance among all subsets, achieving an AUC of 96%, accuracy of 91%, and MCC of 74%. A comprehensive set of results is presented in Table 2 and Figure 3, providing an in-depth analysis of the classification models applied to the original data. These results suggest that despite dataset imbalance, careful feature selection—such as with the 11- and 59-feature sets—can still yield strong predictive performance. However, the use of a propensity score matching process is instrumental in ensuring fair comparisons between the cancer and control groups, significantly contributing to the model’s enhanced accuracy and robustness. 

**Figure 4:**
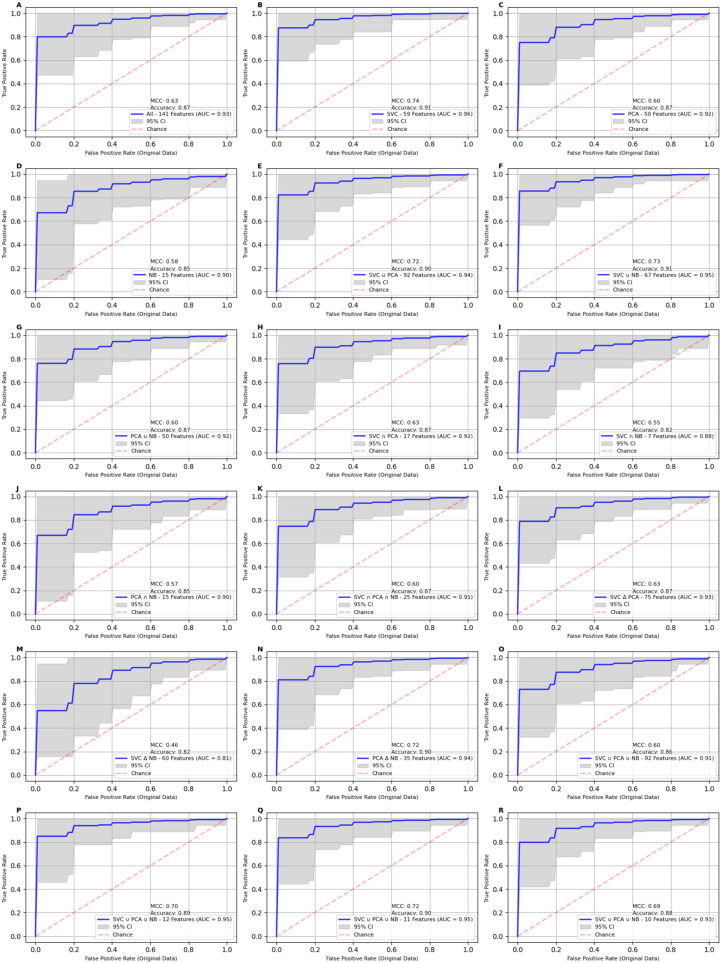
This figure shows the Receiver Operating Characteristic (ROC) curves for classification models applied to the unmatched data. Panels **A**–**R** display AUC curves for the 18 feature sets described in Table 3. When the analysis was restricted to just 54 control cases, performance dropped across all datasets. The feature set in Panel **B** achieved the highest AUC at 96%, followed by those in Panels **F** and **Q**, each at 95%. All panels demonstrate wider confidence intervals and lower performance metrics.

**Figure 5:**
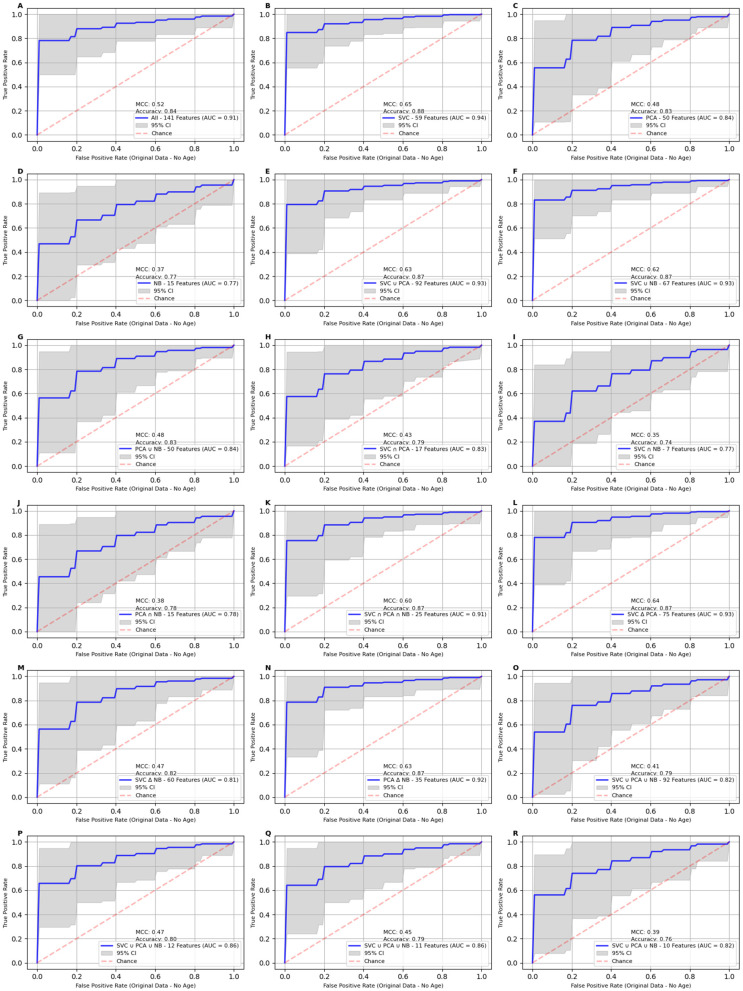
Receiver Operating Characteristic (ROC) curves for classification models applied to the unmatched data after excluding age variable. Panels **A**–**R** display AUC curves for the 18 feature sets described in Table 4. When the analysis was limited to only 54 control cases, performance decreased across all datasets, with notably wide confidence intervals, similar to the PSM dataset after the age variable was excluded. The highest AUC (94%) was achieved by the feature sets in Panels **B** and **F**, while the AUC in Panel **Q** was 10 percentage points lower, reflecting a significant drop in model performance, likely due to the high imbalance between the cancer and control cases.

We found that age, the only demographic feature identified among the 12-, 11-, and 10-feature set is a critical variable in breast cancer prediction. Excluding age from the PSM 11-feature set resulted in a significant reduction in model performance, with the AUC decreasing to 95%, accuracy to 86%, and MCC to 72%, as detailed in Table 3 and Figure 4. This highlights the importance of age as a predictive feature for breast cancer diagnosis and its role in enhancing model performance. The same trend was observed when age was removed from the original dataset. All performance metrics declined significantly, with the AUC dropping to 86%, accuracy falling to 79%, and the MCC decreasing to 45% for the 11-feature set, as shown in Table 4 and Figure 5. This further underscores the critical role of age as a predictive variable. The sharpest decline was observed in feature sets with fewer variables, where more weight is assigned to age as the number of features decreases. Additionally, the risk of developing breast cancer increases as a woman ages [35], further emphasizing the significance of age as a key factor in breast cancer prediction.

Our machine learning framework identified a robust and interpretable set of features for breast cancer prediction, demonstrating strong predictive performance across various feature sets. The 11-feature set emerged as a highly effective subset, balancing model simplicity with high accuracy and AUC values. Propensity score matching was instrumental in addressing the imbalance between cancer and control groups, improving the model’s overall performance. The results indicate that feature selection combined with propensity score matching, is essential for improving model robustness and interpretability when dealing with high-dimensional, imbalanced datasets in breast cancer metabolomics research. 

## 3. Discussion

It is apparent that cancer has a genetic component and, in fact, has been generally accepted as a genetic disease [36]. It is well known that variations in genetic make-up can influence susceptibility to certain diseases including cancer. Furthermore, epigenetic factors (DNA methylation and histone modification) are considered likely to play important roles in the pathogenesis of cancer [37]. Although a number of blood-based cancer assays that detect protein, microRNA, circulating DNA, and methylated DNA biomarkers have been developed, they are specific to late-stage cancer and thus application for screening and/or early detection is rather limited. Furthermore, analytical techniques that require biopsy material for molecular diagnosis are invasive and uncomfortable for the patient. Metabolites and genes are intimately connected [38]. A single DNA base change in a given gene can lead to 10,000-fold shift in the generation of metabolite concentrations that are the products of a sequence of events, i.e., gene transcription, translation, and subsequent protein synthesis and enzyme activation [5,39]. Accordingly, there is an amplification of the signal from DNA to protein to metabolites. It should also be mentioned that there are several factors that can affect the metabolome including ethnicity, sex, age, and diet, as well as geographical location and environment [40]. 

Therefore, there are specific metabolomic signatures that, in conjunction with clinical data, could constitute a panel of biomarkers with vast clinical application for cancer screening. While our work is not meant to de-emphasize the genetic and molecular components of cancer, the field of metabolomic biomarkers is a complimentary field that can be utilized to assist existing screening and surveillance technologies [41]. Metabolomics data presents unique challenges for standard analytical models. The inherent complexity of biomarkers, with its large number of interconnected variables and often limited sample sizes, makes it difficult to identify the most informative markers of disease. This complexity may explain why, despite widespread use of PLS/regression feature selection in metabolomic studies, there remains limited consensus on reliable metabolomic indicators of breast cancer. Our research addressed this by systematically combining multiple machine learning feature selection strategies to derive a small, robust, and reliable panel of biomarkers. 

The 11-feature set comprised of SM(OH) C22:2, SM C18:0, C0, C3OH, C14:2OH, C16:2OH, LysoPC a C18:1, PC aa C36:0, Asparagine, the Kynurenine-to-Tryptophan ratio, and age, achieved an AUC of 98% (CI: 97.5–98%), reflecting a high level of discriminatory power between breast cancer cases and controls. SM(OH) C22:2 and SM C18:0, both sphingomyelins, are involved in maintaining membrane structure, while SM(OH) C16:1 plays a key role in lipid metabolism and membrane integrity [42]. C0 (Carnitine) facilitates the transport of fatty acids for energy production, and C3OH, C14:2OH, and C16:2OH are hydroxycarnitines involved in fatty acid oxidation [43,44]. LysoPC a C18:1 contributes to membrane remodeling, and PC aa C36:0 is a crucial phospholipid within cellular membranes [45]. Asparagine, an essential amino acid, supports protein synthesis [46]. Lastly, the Kynurenine-to-Tryptophan ratio is an important marker for immune regulation and cancer-related inflammation [47].

This performance is comparable to some of the best-performing models found in the literature. For instance, a similar AUC of 98% was achieved in an 11-feature model trained to differentiate between healthy women and patients with four types of solid tumors including breast cancer [13]. A LASSO regression model applied to a subset of 22 biomarkers for triple-negative breast cancer patients achieved an AUC of 96%, slightly lower than our result [14]. Similarly, the panel of seven metabolites related to amino acid metabolism reached an AUC of 80%, which is significantly lower than the AUC values from our feature sets [15]. Another PLS-DA-based model reported an AUC comparing white (AUC = 78%) and African American populations (AUC = 79%), highlighting the impact of demographic variations on model performance [17]. ML models using an ADTree ensemble approach achieved an AUC of 91.2%, which, while competitive, still trails the performance of our model [18]. A model that outperformed our findings used a logistic regression model based on four metabolites, namely N-acetyl-D-tryptophan, 2-arachidonoylglycerol, pipecolic acid, and oxoglutaric acid, and achieved an AUC of 99.5% [28]. However, these results are based on a much smaller serum metabolite dataset of 53 breast cancer patients and 56 controls. 

**Figure 6:**
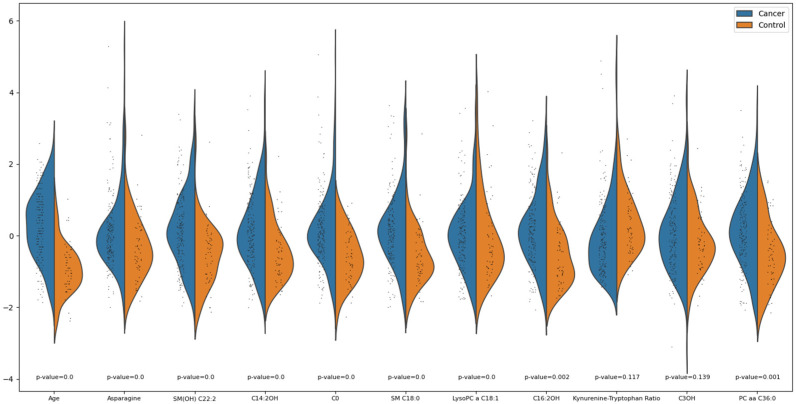
Violin plots of selected features between the cancer and control groups displaying the distribution of feature values for the cancer (blue) and control (orange) groups for 11 selected features. Each violin plot visualizes the kernel density estimate of the feature values, where the width represents the distribution frequency. The *p*-values, computed using the Wilcoxon rank-sum test, are shown below each feature. Features with *p*-values ≤ 0.05 show a statistically significant difference in distribution between the cancer and control groups, whereas the Kynurenine–Tryptophan ratio (*p* = 0.117) and C3OH (*p* = 0.139) display less pronounced statistical significance.

**Figure 7:**
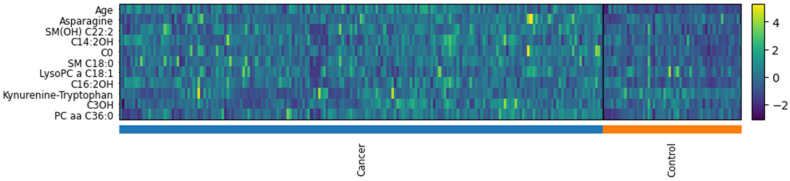
Heatmap of selected 11 features for the cancer and control groups. The color intensity represents the standardized values (Z-scores) of each feature, with the color scale ranging from purple (low expression, −2) to yellow (high expression, +4). The bar at the bottom of the heatmap indicates the sample group assignments, with blue representing cancer and orange representing control. This visual clearly shows the variability in feature expression patterns across samples, highlighting the distinct expression profiles between the two groups.

**Figure 8:**
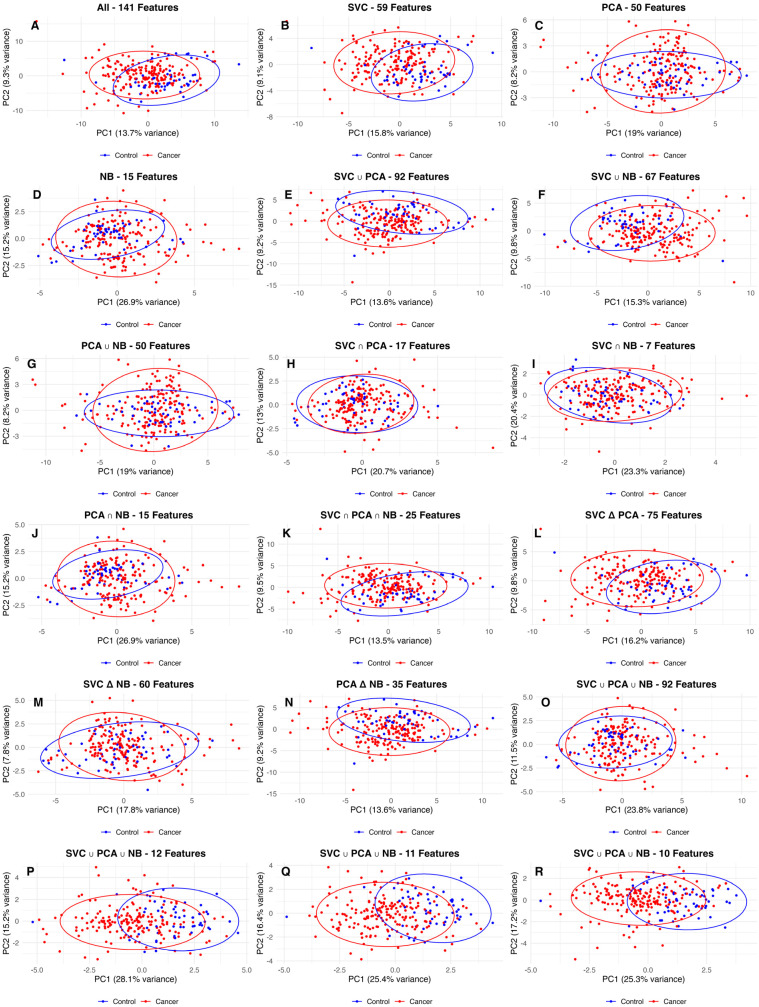
Panels **A**–**R** display PCA visualizations for the 18 feature sets described in Table 3. The 12-, 11-, and 10-feature sets (Panels **P**, **Q** and **R**) exhibit superior separation between control and cancer samples when compared to other feature sets. While other sets, such as SVC–92 and PCA ∩ NB-35 (Panels **B** and **N**) show some separation, refining the feature selection improves the overall distinction between the groups.

In the analysis of differential feature expression between the cancer and control groups, 9 out of the 11 features were identified as having significantly different distributions, as shown in Figure 6. The violin plots provide a detailed visualization of these differences across selected features. The Wilcoxon rank-sum test was employed to assess the statistical significance of these differences, with the Kynurenine-Tryptophan ratio (*p* = 0.117) and C3OH (*p* = 0.139) being the only two features that do not show significant differences. The heatmap displayed in Figure 7 further illustrates the variability in feature expression between the cancer and control groups. The standardized expression values for the selected features were plotted for each sample, providing a clear visualization of the differences across the two groups. The color gradient, representing the Z-scores of each feature, highlights distinct expression patterns, with most of the features showing pronounced differences between the groups. The PCA plots (Figure 8) clearly illustrate that refining the feature set improves the separation between cancer and control samples, particularly in the 11-, 12-, and 10-feature sets.

These reduced feature sets exhibited tighter clustering and clearer division, emphasizing the value of targeted feature selection. This refinement not only improved model performance but also mitigated the risk of overfitting, which is particularly important given the high dimensionality and relatively small sample size of our dataset. The metabolites highlighted in this study as part of the selected 11-feature set that played a critical role in distinguishing between cancer and control groups play critical roles in breast cancer by supporting cancer cell survival and metastasis. These metabolites are relevant to breast cancer because they reflect changes in the key metabolic pathways that cancer cells exploit for growth, survival, immune evasion, and metastasis. 

Machine learning (ML) and deep learning (DL) models have proven particularly adept at handling complex datasets, outperforming traditional methods on highly dimensional data [30,31,48,49,50,51]. Regression, on the other hand, tends to work well on less complex datasets [52]. Our approach addresses the challenges of complex, high-dimensional data and class imbalance more effectively than traditional methods. While many studies rely on singular feature selection techniques like Partial Least Squares (PLS) or LASSO regression, we employed a combination of supervised (SVC, Naive Bayes) and unsupervised (PCA) techniques, resulting in more robust and interpretable feature selection. Additionally, our use of propensity score matching (PSM) to balance the cancer and control groups provides more reliable and unbiased results, a practice not commonly applied in metabolomics studies. Furthermore, we incorporated a random sampling strategy and explored the unions and intersections of feature sets from multiple methods, ensuring the consistent identification of the most predictive biomarkers.

The robust performance of our 11-variable panel lays the groundwork for future research and clinical applications. Firstly, this metabolomic signature could be developed into a rapid, non-invasive blood test for breast cancer screening, potentially complementing or even preceding mammography in certain populations. Secondly, the identified biomarkers might prove valuable for risk stratification, monitoring treatment response and disease progression, enabling more personalized patient management. Additionally, this metabolomic approach could be extended to investigate its utility in distinguishing between different breast cancer subtypes. Finally, similar approaches could be applied to other cancer types, potentially improving cancer screening across multiple malignancies. Despite these strengths, we share some limitations with other studies, namely a relatively small sample size. Future large-scale, prospective studies will be crucial to validate these applications and fully realize the potential of metabolomic profiling in oncology.

## 4. Materials and Methods

### 4.1. Study Samples

A total of 185 prospectively collected archived plasma samples from women with biopsy-confirmed breast cancer and 53 plasma samples from healthy controls were obtained from the Cooperative Health Tissue Network (CHTN) biobank (National Institutes of Health, National Cancer Institute, Bethesda, MD, USA) From a histological standpoint, the cancer cases consisted of 41 lobular carcinoma samples and 144 ductal carcinoma samples. Nearly 90% of the cancer patients were in stages I (98 patients) and II (70 patients), while the remaining 17 patients were classified as stage III.

### 4.2. Analytical Procedures

A targeted, quantitative mass spectrometry (MS)-based metabolomics approach was undertaken to analyze 138 metabolites in the plasma samples by DI-LC/MS/MS using the TMIC (The Metabolomics Innovation Centre, Edmonton, AB, Canada) PRIME assay as previously described [29,53]. Mass spectrometric analysis of the diluted extracts was performed on an HPLC (Agilent 1260 HPLC, Agilent Technologies, Santa Clara, CA, USA) equipped with a Qtrap^®^ 4000 tandem mass spectrometry instrument (Applied Biosystems/MDS Analytical Technologies, Foster City, CA, USA). This assay enables the targeted identification and quantification of up to 138 different endogenous metabolites, including amino acids, acylcarnitines, biogenic amines and derivatives, organic acids, uremic toxins, glycerophospholipids, sphingolipids, and sugars. The method employs chemical derivatization (via 3-NPH for organic acids or PITC for amine-containing compounds), analyte extraction and separation, and selective mass-spectrometric detection using multiple reaction monitoring (MRM) pairs for metabolite identification and quantification. Isotope-labeled ISTDs (internal standard spiking solution), along with other ISTDs are used for accurate metabolite quantification.

### 4.3. Satistical Analysis

The recommended statistical procedures for standard quantitative metabolomic analysis were followed as previously outlined in prior studies [54,55]. The dataset was divided into a training set (80%) and a validation set (20%). In addition to metabolite concentration data, demographic data was utilized to identify optimal biomarker sets. For each subgroup, performance metrics were computed, including the area under the receiver operating characteristic (AUC) curves, precision, recall, Matthew’s correlation coefficient, F1-score, and overall accuracy. As part of the data preprocessing steps, male subjects were excluded from the breast cancer dataset. Variables with more than 30% missing values were also removed, resulting in the retention of 132 biomarkers. For the remaining variables, missing values were imputed with the instrument detection limit for metabolites, and demographic data were filled with mean values. The demographic variables included age, BMI, race, and smoking status. Smoking status was one-hot encoded into categories: current smoker, past smoker, and smoking history. Race was categorized into white, African American, and other. Additionally, a metabolite ratio, specifically the Kynurenine-to-Tryptophan ratio, was added to the dataset, as this ratio is believed to be a strong indicator in the development of the psychoneurological symptoms associated with breast cancer [47]. The final dataset comprised 141 features, of which 132 were biomarkers, 1 a metabolite ratio, and 8 were demographic variables as described. Continuous data were then subjected to standard normalization techniques.

## 5. Conclusions

The multi-step methodology employed in this study, integrating a multi-model feature selection strategy with propensity score matching resulted in a robust, interpretable, and highly predictive model for breast cancer detection. The strengths of our approach lie in its potential to address the limitations of traditional breast cancer screening. Metabolomic profiling offers the potential for a non-invasive way to detect subtle, early-stage cancer signatures. Additionally, the small, robust biomarker panel facilitates a more cost-effective and interpretable approach than analyses involving large numbers of variables. Additionally, novel features for breast cancer, such as the identified biomarkers related to immune suppression and metabolism, offer promising avenues for non-invasive diagnostics. While our results are highly promising, the relatively small sample size highlights the need for larger validation studies. For practical applications, this methodology could be expanded and validated on larger datasets, such as those from clinical settings or diverse patient populations, to confirm its robustness and generalizability. Despite the sample size limitation, our work demonstrates the power of a targeted, multi-pronged analytical approach for identifying metabolic markers in breast cancer. Similar methods, including the machine learning models applied to metabolomics, have shown potential in breast cancer research, further supporting the viability of our approach for clinical implementation. This research provides a strong foundation for developing more sensitive and accessible cancer screening tools.

## Figures and Tables

**Table 1:** Support Vectors Classifier (SVC) results on propensity-score-matched (PSM) datasets.

PSM Data							
Feature Set	AUC		Accuracy	MCC	F1 Score	Precision	Recall
All-141 Feat	99%	(CI: 98.4–98.8%)	93%	85%	95%	96%	93%
SVC-59 Feat	99%	(CI: 99.1–99.4%)	95%	90%	95%	97%	94%
PCA-50 Feat	98%	(CI: 98.1–98.5%)	94%	87%	95%	97%	94%
NB-15 Feat	97%	(CI: 96.3–96.9%)	91%	81%	95%	97%	93%
SVC ∪ PCA-92 Feat	99%	(CI: 98.7–99.1%)	95%	90%	95%	97%	93%
SVC ∪ NB-67 Feat	99%	(CI: 99.1–99.3%)	95%	90%	95%	97%	94%
PCA ∪ NB-50 Feat	98%	(CI: 97.8–98.3%)	94%	87%	95%	97%	94%
SVC ∩ PCA-17 Feat	97%	(CI: 97.1–97.7%)	93%	85%	95%	97%	93%
SVC ∩ NB-7 Feat	95%	(CI: 94.2–94.9%)	86%	72%	94%	97%	92%
PCA ∩ NB-15 Feat	96%	(CI: 96.3–96.9%)	91%	81%	94%	97%	92%
SVC ∩ PCA ∩ NB-25 Feat	98%	(CI: 98.4–98.8%)	94%	87%	94%	97%	92%
SVC Δ PCA-75 Feat	99%	(CI: 98.4–98.8%)	94%	87%	94%	97%	92%
SVC Δ NB-60 Feat	96%	(CI: 95.5–96.5%)	91%	82%	94%	97%	92%
PCA Δ NB-35 Feat	99%	(CI: 98.8–99.2%)	95%	89%	94%	97%	92%
SVC ∪ PCA ∪ NB-92 Feat	98%	(CI: 97.3–97.9%)	93%	85%	94%	97%	92%
SVC ∪ PCA ∪ NB-12 Feat	98%	(CI: 97.2–97.8%)	94%	89%	94%	97%	92%
SVC ∪ PCA ∪ NB-11 Feat	98%	(CI: 97.5–98.0%)	95%	89%	94%	97%	92%
SVC ∪ PCA ∪ NB-10 Feat	97%	(CI: 97.3–97.8%)	93%	85%	94%	97%	92%

**Table 2:** Support Vectors Classifier (SVC) results on propensity-score-matched (PSM) datasets after excluding the age variable.

PSM Data (Excluding Age)							
Feature Set	AUC		Accuracy	MCC	F1 Score	Precision	Recall
All-141 Feat	98%	(CI: 98.1–98.5%)	92%	83%	94%	96%	92%
SVC-59 Feat	99%	(CI: 99.0–99.3%)	94%	88%	95%	97%	93%
PCA-50 Feat	96%	(CI: 96.0–96.8%)	92%	84%	94%	96%	93%
NB-15 Feat	94%	(CI: 93.6–94.5%)	87%	74%	93%	96%	91%
SVC ∪ PCA-92 Feat	99%	(CI: 98.6–98.9%)	94%	87%	94%	96%	91%
SVC ∪ NB-67 Feat	99%	(CI: 98.8–99.1%)	93%	87%	94%	96%	92%
PCA ∪ NB-50 Feat	97%	(CI: 96.3–97.0%)	92%	84%	94%	96%	92%
SVC ∩ PCA-17 Feat	95%	(CI: 94.3–95.2%)	88%	76%	93%	96%	91%
SVC ∩ NB-7 Feat	89%	(CI: 88.1–89.3%)	81%	62%	92%	96%	89%
PCA ∩ NB-15 Feat	94%	(CI: 93.5–94.4%)	87%	74%	92%	96%	89%
SVC ∩ PCA ∩ NB-25 Feat *	99%	(CI: 98.4–98.8%)	94%	87%	92%	96%	89%
SVC Δ PCA-75 Feat *	99%	(CI: 98.5–98.9%)	94%	87%	93%	96%	90%
SVC Δ NB-60 Feat *	96%	(CI: 95.5–96.4%)	91%	82%	93%	96%	90%
PCA Δ NB-35 Feat	99%	(CI: 98.6–99.0%)	94%	87%	93%	96%	90%
SVC ∪ PCA ∪ NB-92 Feat	96%	(CI: 95.0–95.9%)	88%	76%	93%	96%	90%
SVC ∪ PCA ∪ NB-12 Feat	94%	(CI: 93.9–94.7%)	86%	73%	92%	96%	89%
SVC ∪ PCA ∪ NB-11 Feat	95%	(CI: 93.7–94.5%)	86%	72%	92%	96%	89%
SVC ∪ PCA ∪ NB-10 Feat	92%	(CI: 91.8–92.8%)	84%	70%	92%	96%	89%

* Age not included in this subset.

**Table 3:** Support Vectors Classifier (SVC) results on original (unmatched) datasets.

Original Data							
Feature Set	AUC		Accuracy	MCC	F1 Score	Precision	Recall
All-141 Feat	93%	(CI: 91.8–92.8%)	87%	63%	92%	91%	94%
SVC-59 Feat	96%	(CI: 95.2–96.0%)	91%	74%	93%	92%	94%
PCA-50 Feat	92%	(CI: 91.0–92.0%)	87%	60%	93%	91%	94%
NB-15 Feat	90%	(CI: 89.1–90.4%)	85%	58%	92%	91%	93%
SVC ∪ PCA-92 Feat	94%	(CI: 93.6–94.5%)	90%	72%	92%	92%	93%
SVC ∪ NB-67 Feat	95%	(CI: 94.5–95.3%)	91%	73%	93%	92%	94%
PCA ∪ NB-50 Feat	92%	(CI: 90.9–91.9%)	87%	60%	92%	92%	94%
SVC ∩ PCA-17 Feat	92%	(CI: 90.9–91.9%)	87%	63%	92%	92%	93%
SVC ∩ NB-7 Feat	88%	(CI: 87.0–88.3%)	82%	55%	92%	92%	92%
PCA ∩ NB-15 Feat	90%	(CI: 89.1–90.3%)	85%	57%	92%	92%	92%
SVC ∩ PCA ∩ NB-25 Feat	91%	(CI: 90.6–91.8%)	87%	60%	92%	91%	92%
SVC Δ PCA-75 Feat	93%	(CI: 92.2–93.2%)	87%	63%	92%	91%	92%
SVC Δ NB-60 Feat	81%	(CI: 79.7–81.7%)	82%	46%	91%	91%	92%
PCA Δ NB-35 Feat	94%	(CI: 93.6–94.5%)	90%	72%	92%	91%	92%
SVC ∪ PCA ∪ NB-92 Feat	91%	(CI: 90.3–91.5%)	86%	60%	92%	91%	92%
SVC ∪ PCA ∪ NB-12 Feat	95%	(CI: 94.1–95.0%)	89%	70%	92%	91%	92%
SVC ∪ PCA ∪ NB-11 Feat	95%	(CI: 94.2–95.0%)	90%	72%	92%	92%	92%
SVC ∪ PCA ∪ NB-10 Feat	93%	(CI: 92.6–93.6%)	88%	69%	92%	92%	92%

**Table 4:** Support Vectors Classifier (SVC) results on original (unmatched) datasets after excluding the age variable.

Original Data (Excluding Age)							
Feature Set	AUC		Accuracy	MCC	F1 Score	Precision	Recall
All-141 Feat	91%	(CI: 90.4–91.5%)	84%	52%	90%	88%	92%
SVC-59 Feat	94%	(CI: 94.0–94.9%)	88%	65%	91%	90%	93%
PCA-50 Feat	84%	(CI: 83.2–84.8%)	83%	48%	91%	89%	93%
NB-15 Feat	77%	(CI: 76.3–78.1%)	77%	37%	89%	88%	91%
SVC ∪ PCA-92 Feat	93%	(CI: 91.9–92.8%)	87%	63%	90%	89%	91%
SVC ∪ NB-67 Feat	93%	(CI: 92.9–93.8%)	87%	62%	90%	89%	92%
PCA ∪ NB-50 Feat	84%	(CI: 83.0–84.7%)	83%	48%	90%	89%	92%
SVC ∩ PCA-17 Feat	83%	(CI: 82.5–84.0%)	79%	43%	90%	89%	91%
SVC ∩ NB-7 Feat	77%	(CI: 75.6–77.7%)	74%	35%	89%	89%	89%
PCA ∩ NB-15 Feat	78%	(CI: 76.5–78.4%)	78%	38%	88%	88%	89%
SVC ∩ PCA ∩ NB-25 Feat *	91%	(CI: 90.5–91.6%)	87%	60%	89%	89%	89%
SVC Δ PCA-75 Feat *	93%	(CI: 92.0–93.0%)	87%	64%	89%	89%	90%
SVC Δ NB-60 Feat *	81%	(CI: 79.9–81.8%)	82%	47%	89%	89%	90%
PCA Δ NB-35 Feat	92%	(CI: 91.7–92.8%)	87%	63%	89%	89%	90%
SVC ∪ PCA ∪ NB-92 Feat	82%	(CI: 81.0–82.8%)	79%	41%	89%	89%	90%
SVC ∪ PCA ∪ NB-12 Feat	86%	(CI: 85.2–86.7%)	80%	47%	89%	89%	89%
SVC ∪ PCA ∪ NB-11 Feat	86%	(CI: 85.2–86.7%)	79%	45%	89%	89%	89%
SVC ∪ PCA ∪ NB-10 Feat	82%	(CI: 81.2–82.8%)	76%	39%	88%	89%	89%

* Age not included in this subset.

## Data Availability

Data is unavailable due to privacy or ethical restrictions.
